# A Case of Laryngeal Diphtheria in a Nursing Baby Three Months Old

**Published:** 1906-01

**Authors:** Lynn S. Beals

**Affiliations:** Buffalo, N. Y.


### A case of Laryngeal Diphtheria in a Nursing Baby Three Months Old.

BY LYNN S. BEALS, M. D., Buffalo, N. Y.

A Case of Laryngeal Diptheria in a Nursing baby 3 mos. old.

Paul S——, 81 P—— St., age 3 mos. 26 days, nursing exclusively from breast.

First seen November 10, 1905. Mother said that child had had a cough for three days, worse at night, that child was getting better, but that she was worried somewhat. Nursed all right and slept well, save for a slight coughing at night.

Child did not look sick. Temperature 98°. Pulse 144. Respiration 88 (crying). A few fine râles in left posterior base; a very slight redness of pharynx, otherwise physical examination negative. Breathing harsh, and rapid out of proportion to findings. No retraction of epigastrium or interpaces. Culture taken. November 11, 1905, report received—Positive for K. L. 3000 units immediately given into right thigh.

Warning was given for no one to kiss the baby, whereupon the mother admonished Earl, her son of 7, not to kiss the baby *any more.* Earl was then noticed to have a unitateral irritating discharge from nose, with no membrane. This nasal discharge he had had for two weeks, going to school during that time. Culture taken from nasal secretion. November 12, 1905, report received, positive for K. L. Earl then given 3000 units.

Paul quickly improved, and on the 12th had no symptoms, and on the 24th of November culture was negative.

Earl gave a negative culture on the 21st of November. The mother was given 500 units at the outset and nursing continued, with local cleansing before and after nursing; mother did not acquire diptheria.

177 DEARBORN STREET.

